# Mutational Analysis of the Rift Valley Fever Virus Glycoprotein Precursor Proteins for Gn Protein Expression

**DOI:** 10.3390/v8060151

**Published:** 2016-05-24

**Authors:** Inaia Phoenix, Nandadeva Lokugamage, Shoko Nishiyama, Tetsuro Ikegami

**Affiliations:** 1 Department of Pathology, The University of Texas Medical Branch, Galveston, TX 77555, USA; inphoeni@UTMB.EDU (I.P.); nalokuga@UTMB.EDU (N.L.); shnishiy@UTMB.EDU (S.N.); 2 The Sealy Center for Vaccine Development, The University of Texas Medical Branch, Galveston, TX 77555, USA; 3 The Center for Biodefense and Emerging Infectious Diseases, The University of Texas Medical Branch, Galveston, TX 77555, USA

**Keywords:** Rift Valley fever virus, M-segment, Gn, 78 kD, NSm, precursor, expression strategy, reporter assay, reverse genetics

## Abstract

The Rift Valley fever virus (RVFV) M-segment encodes the 78 kD, NSm, Gn, and Gc proteins. The 1st AUG generates the 78 kD-Gc precursor, the 2nd AUG generates the NSm-Gn-Gc precursor, and the 3rd AUG makes the NSm’-Gn-Gc precursor. To understand biological changes due to abolishment of the precursors, we quantitatively measured Gn secretion using a reporter assay, in which a *Gaussia* luciferase (gLuc) protein is fused to the RVFV M-segment pre-Gn region. Using the reporter assay, the relative expression of Gn/gLuc fusion proteins was analyzed among various AUG mutants. The reporter assay showed efficient secretion of Gn/gLuc protein from the precursor made from the 2nd AUG, while the removal of the untranslated region upstream of the 2nd AUG (AUG2-M) increased the secretion of the Gn/gLuc protein. Subsequently, recombinant MP-12 strains encoding mutations in the pre-Gn region were rescued, and virological phenotypes were characterized. Recombinant MP-12 encoding the AUG2-M mutation replicated slightly less efficiently than the control, indicating that viral replication is further influenced by the biological processes occurring after Gn expression, rather than the Gn abundance. This study showed that, not only the abolishment of AUG, but also the truncation of viral UTR, affects the expression of Gn protein by the RVFV M-segment.

## 1. Introduction

Rift Valley fever (RVF) is a mosquito-borne zoonotic disease affecting humans and ruminants. The disease was originally endemic to sub-Saharan Africa, but it has since spread to Egypt, Madagascar, Saudi Arabia, and Yemen [1,2]. RVF causes a high-rate of abortion in sheep, cattle, and goats, and hemorrhagic fever, encephalitis, or blindness in humans [3]. The mortality rate of RVF patients is considered to be less than 0.5% to 1% [3,4]. However, RVF outbreaks have been known to involve a large number of patients: For example, 20,000 to 200,000 infections and 600 deaths in Egypt in 1977–1978. Floodwater *Aedes* mosquitoes can transovarially transmit RVFV [5]. These eggs are resistant to draught, and flooding, due to heavy rainfall, facilitates the hatching of infected eggs [1]. RVF is an important public health and agricultural concern, and vaccination of susceptible animals is important to minimize the spread of disease [6]. Though live-attenuated RVF vaccines are available for veterinary use in endemic countries, RVF outbreaks still occur in Africa and the surrounding countries, indicating the requirement of more effective control measures.

**Figure 1:**
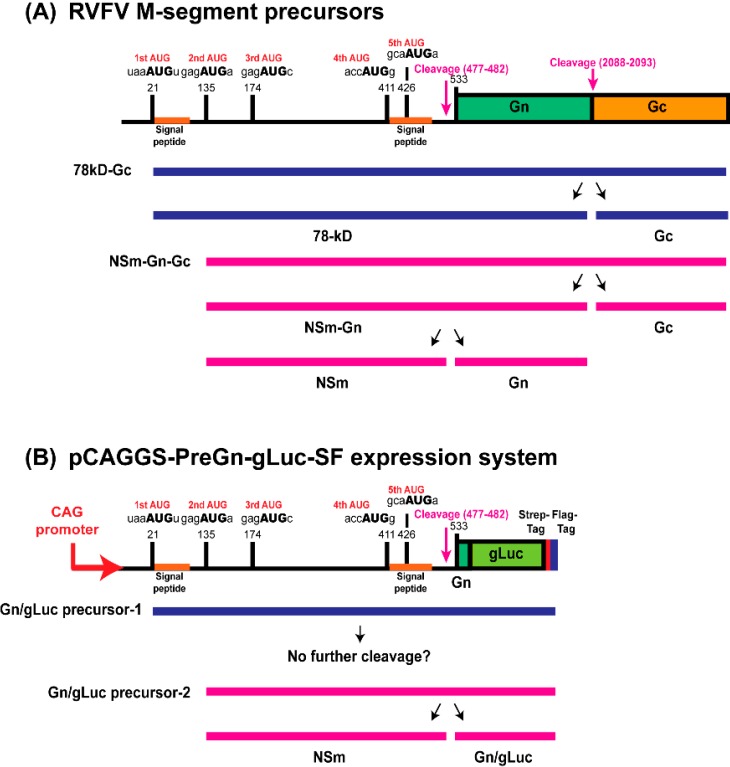
Gene expression of the RVFV M-segment and pCAGGS-PreGn-gLuc-SF. (**A**) The polypeptides synthesized from the 1st AUG (78 kD-Gc) or the 2nd AUG (NSm-Gn-Gc) are cleaved by signal peptidases [14,16,28]. The 78 kD protein and Gc are generated from the 78 kD-Gc precursor, while NSm, Gn, and Gc are made from the NSm-Gn-Gc precursor; (**B**) the pre-Gn region was fused to the gLuc ORF lacking the intrinsic signal peptide, which allows for secretion from the cell via Gn signal peptide. The Gn/gLuc precursor-1 makes a chimeric protein consisting of the pre-Gn region and gLuc, while the Gn/gLuc precursor-2 generates NSm and the Gn/gLuc fusion protein.

Rift Valley fever virus (RVFV: genus *Phlebovirus*, family *Bunyaviridae*) is comprised of three segmented, negative-stranded RNA: the Large (L), Medium (M), and Small (S)-segments. The L-segment encodes the RNA-dependent RNA polymerase (L protein). The S-segment encodes nucleocapsid (N) and nonstructural S (NSs) proteins in an ambi-sense manner, and the NSs protein is dispensable for viral replication. However, the NSs serves as the major virulence factor for RVF, and counteracts host antiviral responses by shutting-off host general transcription, including the interferon (IFN)-β gene, and promoting the posttranslational degradation of transcription factor (TF)IIH p62 subunits and dsRNA-dependent protein kinase (PKR) [7,8,9,10,11,12,13]. The M-segment encodes the envelope glycoproteins Gn and Gc, and two accessory proteins, NSm and 78 kD [14,15,16]. The Kozak consensus sequence (5′-**G**CC **R**CC AUG **G**-3′) affects translation initiation efficiency, through the nucleotides located at the −6, −3 and +4 positions in vertebrate cells [17]. In the RVFV M mRNA, the first AUG is surrounded by a weak Kozak context (5′-CAU UAA AUG U-3′), for vertebrate cells [18]. On the other hand, the 2nd AUG partially matches the Kozak context (5′-CCA **G**AG AUG A) via the guanosine at the −3 position. As a result, RVFV M-segment allows a leaky scanning of the ribosome at the 1st AUG [17]. Thus, two polypeptides can be generated using those two initiation codons starting at nt. 21 (1st AUG) and 135 (2nd AUG) (Figure 1A). The precursor protein from the 1st AUG is cleaved to produce 78 kD and Gc, while the precursor from the 2nd AUG is cleaved to produce NSm, Gn, and Gc. When the 2nd AUG is abolished, the precursor from the 3rd AUG generate NSm’, Gn, and Gc [18]. Since the 3rd, 4th, or 5th AUG are also able to generate Gn-Gc precursors, the 2nd AUG can be abolished without affecting the expression of Gn and Gc.

The live-attenuated RVF MP-12 vaccine is safe and efficacious in ruminants, and is conditionally licensed for veterinary use in the U.S. [19,20,21,22,23,24]. However, the MP-12 vaccine lacks a marker for the differentiation of infected from vaccinated animals (DIVA). The NSm and 78 kD proteins are dispensable for viral replication [25,26]. Based on the strong immunogenicity, a recombinant MP-12 vaccine encoding an in-frame truncation in the 78 kD/NSm region in the M-segment (rMP12-ΔNSm21/384) is considered as one of the next generation of MP-12 vaccines. The rMP12-ΔNSm21/384 lacking both 78 kD and NSm induces apoptosis earlier than parental rMP-12 in Vero E6, 293, and J774.1 cells [25]. In another study, the C-terminal (aa. 71–115) region of the NSm protein, which overlaps with NSm’, a truncated NSm generated from the 3rd AUG, was shown to be sufficient to suppress apoptosis [27]. NSm and NSm’ localize to the mitochondrial outer membrane, through the C-terminal transmembrane domain [18,27]. Thus, an rMP-12 strain lacking the 78 kD and NSm proteins would be a viable candidate vaccine as it would have good immunogenicity and a DIVA marker.

Since the 78 kD and NSm/NSm’ proteins are synthesized from distinct precursor polyproteins made from the 1st and 2nd AUG (*i.e.*, 78 kD-Gc and NSm-Gn-Gc, respectively), the alteration of the 1st or 2nd AUG may also affect the synthesis of Gn or Gc. Using recombinant vaccinia viruses, Suzich *et al.* analyzed the impact of specific AUG abolishment (*i.e.*, AUG to CUC substitution) in the pre-Gn region on Gn expression levels. The abolishment of the 1st AUG (Δ1) slightly increased the Gn expression, whereas the abolishment of the 2nd AUG (Δ2), the 2nd and 3rd AUGs (Δ2 + 3), the 2nd, 3rd, and 4th AUGs (Δ2 + 3 + 4), or the 2nd, 3rd, 4th and 5th AUGs (Δ2 + 3 + 4 + 5) decreased the accumulation of Gn, compared to that from the parental wild-type M-segment. Thus, the “default” NSm-Gn-Gc precursor protein from the 2nd AUG is apparently more efficient than the NSm’-Gn-Gc precursor (from the 3rd AUG) or the Gn-Gc precursors (from the 4th or 5th AUGs), for Gn expression. However, further quantitative analysis of Gn expression changes by AUG alterations will be required to correctly understand the impact of mutagenesis in the M-segment start codons.

Little information is available about the consequence of pre-Gn region mutagenesis in terms of viral phenotypes, other than the expression of 78 kD or NSm/NSm’. We hypothesized that an optimization of the M-segment pre-Gn region increases the secretion of Gn, which in turn, will lead to an increase in viral titer. We aimed to analyze the consequences of the abolishment of the 1st, 2nd, 3rd, 4th, and/or 5th AUG, in terms of Gn secretion and viral phenotype. We established a reporter assay, in which *Gaussia* luciferase (gLuc), fused to the N-terminal region of Gn, is expressed from the pre-Gn region of the RVFV M-segment. In the reporter assay, the Gn/gLuc fusion protein is cleaved from the Gn/gLuc precursor proteins made from either the 1st or 2nd AUG, while the 3rd, 4th, or 5th AUG can serve as surrogates for precursor production, as shown in Figure 1B. Normally, gLuc encodes an intrinsic signal sequence, which allows it to be secreted from expressed cells [29]. In our reporter assay, we deleted the intrinsic signal sequence for gLuc so that the cleavage of Gn/gLuc fusion proteins occurs only through the Gn signal sequence, and subsequently, the Gn/gLuc fusion proteins are secreted from expressed cells. The Gn/gLuc fusion proteins produced from this construct do not accumulate in the Golgi, due to a lack of the Golgi retention signal at the C-terminus of Gn [30], and the secretion occurs through the endocytic recycling pathway from the Golgi to plasma membrane (e.g., Rab8-positive vesicles) [31]. Although the level of Gn/gLuc fusion protein secretion does not predict virion release efficiency, as RVFV Gn/Gc accumulates in the Golgi, we aimed to measure relative expression level of Gn protein from precursor proteins made from RVFV M-segment. We analyzed the relative secretion of the Gn/gLuc fusion proteins into the culture supernatant among different AUG (Met) to CUC (Leu) substitution mutants. Furthermore, recombinant MP-12 (rMP-12) encoding the AUG mutations, which can secrete distinct levels of Gn, were rescued by reverse genetics, and their phenotypes were characterized. Our study provides fundamental information for the consequences of mutagenesis in the pre-Gn region of the M-segment, and will support the understanding of current and future studies using NSm or 78 kD knockout mutants.

## 2. Materials and Methods

### 2.1. Media, Cells, and Viruses

Human embryonic kidney (293) cells, Vero cells, (ATCC CCL-81), and Vero E6 cells (ATCC C1008) were maintained in Dulbecco’s Modified Eagle Medium (DMEM) supplemented with 10% FBS, penicillin (100 U/mL), and streptomycin (100 μg/mL). Minimum Essential Medium (MEM)-alpha supplemented with 10% fetal bovine serum (FBS) (Life Technologies, Carlsbad, CA, USA), penicillin (100 U/mL), streptomycin (100 μg/mL), and hygromycin B (600 μg/mL) was used to culture BHK/T7-9 cells that stably express T7 RNA polymerase [32]. Recombinant RVFV MP-12 strains encoding mutation(s) in the M-segment pre-Gn region were rescued by reverse genetics, as described previously [33]. rMP-12 mutants were titrated by plaque assay using Vero E6 cells [34].

### 2.2. Plasmids

The pCAGGS-PreGn-gLuc-SF plasmid was made as follows: Synthetic DNA was created (gBlocks, Integrated DNA Technologies, Coralville, IA, USA) for the RVFV MP-12 M-segment (nt. 1-533) fused with gLuc (lacking nt.1-51 to remove an intrinsic signal sequence for gLuc), and two tandem Strep-tags, and a Flag-tag. The DNA fragment was cloned into pCAGGS plasmid by Gibson assembly mastermix (New England BioLabs, Ipswich, MA, USA). To introduce mutations in pCAGGS-PreGn-gLuc-SF plasmid, the DNA fragment (nt. 1-830) was first transferred to a pProT7 plasmid. Then, site-directed mutagenesis was performed using the pProT7 plasmid back-bone, before cloning the insert sequence into a pCAGGS plasmid. Corresponding AUGs were replaced with CUC (Leu) to abolish the AUG codon.

### 2.3. Western Blotting

Cells were suspended in 2x SDS sample buffer, and boiled for 10 min. Samples were separated by SDS-polyacrylamide gel electrophoresis (SDS-PAGE) under reducing conditions. Western blot was performed as described previously [35]. Anti-Flag M2 antibody (Sigma-Aldrich, St. Louis, MO, USA), and anti-actin antibody (I-19, Santa Cruz Biotechnology, Inc., Dallas, TX, USA) were used.

### 2.4. The Gaussia and Cypridia Luciferase Assays

Sub-confluent 293 cells (1 × 10^6^ cells) were mock-transfected or co-transfected with 0.1 μg of pSV40-CLuc (encoding *Cypridia* luciferase, cLuc, downstream of SV40 promoter) and 2.0 μg of pCAGGS-PreGn-gLuc-SF, or the mutants in the pre-Gn region. At 36 h post transfection, culture supernatants were harvested, and gLuc assays (BioLuc Gaussia Luciferase Assay Kit, New England BioLabs) and cLuc assays (BioLuc Cypridina Luciferase Assay Kit, New England BioLabs) were performed, according to manufacturer’s instructions.

### 2.5. Measurement of Plaque Sizes

Plaque images of rMP-12, Δ2 + 3, or the AUG2-M mutants, formed in VeroE6 cells in 6-well plates, were incorporated by a scanner, and the diameters (mm) of small and large plaques (*n* = 10 each) were measured using Adobe Photoshop Element version 7.0 [36]. The average and standard errors were plotted onto the graph using GraphPad Prism version 6.05 [37].

### 2.6. Statistical Analysis

**Figure 2:**
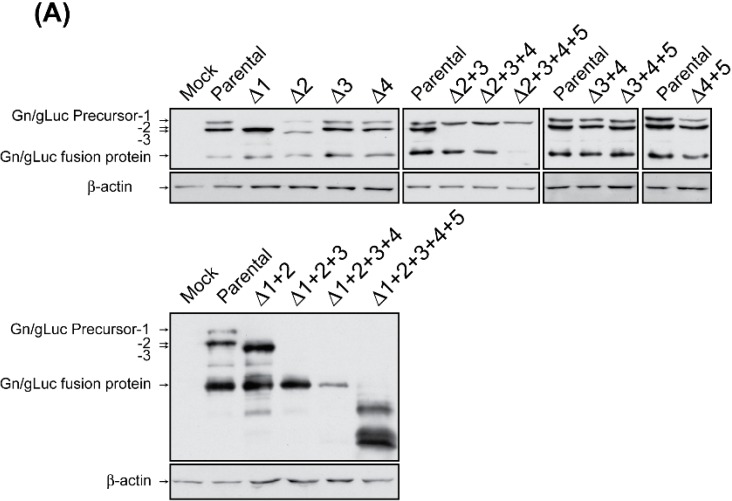

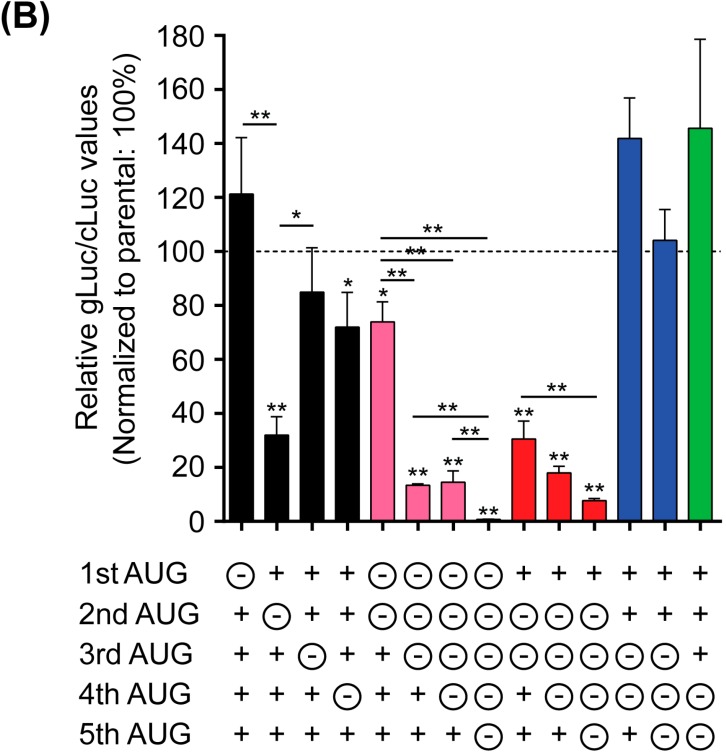
Relative expression of precursor proteins and cleaved Gn/gLuc fusion proteins after AUG abolishment. (**A**) Western blot of cell lysates. 293 cells were co-transfected with pSV40-CLuc (transfection control), and pCAGGS-PreGn-gLuc-SF or the AUG mutant plasmids. At 36 h post transfection, cell lysates were collected and analyzed by Western blot using anti-Flag antibody; (**B**) the extracellular Gn/gLuc fusion proteins were measured using the culture supernatant of transfected cells. The ratio of gLuc to cLuc (control plasmid) was normalized to that of parental pCAGGS-PreGn-gLuc-SF. The graph represents the mean + the standard error of three independent experiments. Asterisks represent statistically significant differences (one-way ANOVA followed by Tukey’s multiple comparisons test, * *p* < 0.05, ** *p* < 0.01). Asterisks shown on error bars represent the comparison with compared to the Δ4 + 5 mutant.

**Figure 3:**
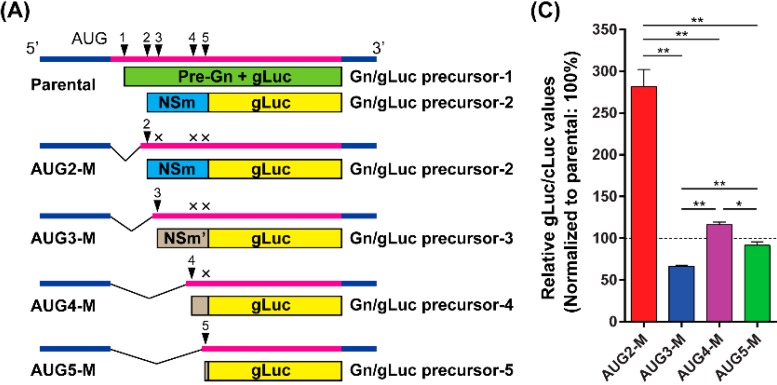

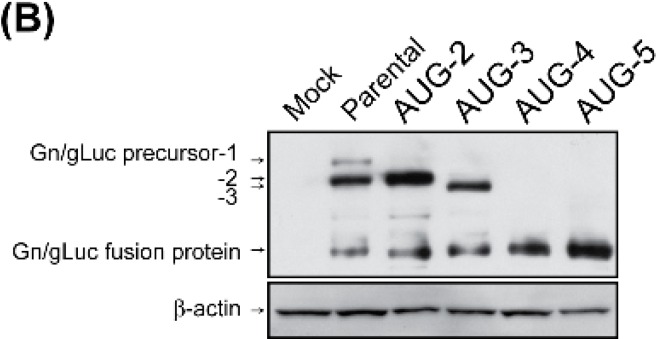
Relative expression of precursor proteins and cleaved gLuc after the truncation of the viral sequence upstream of AUG. (**A**) Schematic representation of AUG2-M, AUG3-M, AUG4-M, or AUG5-M, which express a single precursor from the 2nd, 3rd, 4th, or the 5th AUG, respectively. AUG2-M lacks the 3rd, 4th, and 5th AUGs, AUG3-M lacks the 4th and 5th AUGs, and AUG4-M lacks the 5th AUG. The mutant plasmids encode a common 20 nucleotide viral sequence upstream of the 1st AUG (5′-ACACAAAGACGGUGCACGAGAUG (initiation codon is underlined)); (**B**) 293 cells were co-transfected with pSV40-CLuc (transfection control), and pCAGGS-PreGn-gLuc-SF or the mutant plasmids. At 36 h post transfection, culture supernatants were collected, and the gLuc and cLuc activities were measured. Then, the ratio of gLuc to cLuc was normalized to that of parental pCAGGS-PreGn-gLuc-SF plasmid. The graph represents the mean + the standard error of three independent experiments. Asterisks represent statistically significant differences (one-way ANOVA followed by Tukey’s multiple comparisons test, * *p* < 0.05, ** *p* < 0.01).

**Figure 4:**
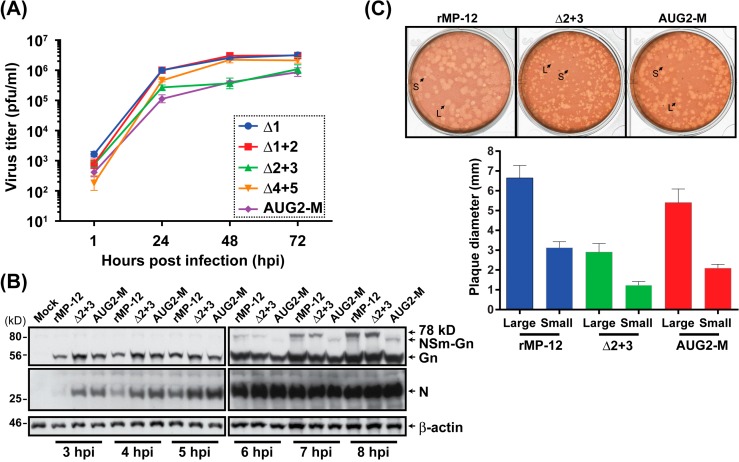
Characterization of recombinant MP-12 encoding mutations in the pre-Gn region. (**A**) Replication of rMP-12 mutants encoding the mutations either in the 1st AUG (Δ1), the 1st and 2nd AUG (Δ1 + 2), the 2nd and 3rd AUG (Δ2 + 3), the 4th and 5th AUG (Δ4 + 5), or the deletion of the UTR upstream of the 2nd AUG (AUG2-M: see Figure 3A). Vero cells were infected with each virus at a multiplicity of infection (MOI) of 0.15. The graph represents the antilog of the arithmetic mean of the log_10_-transformed virus titers + the standard deviation of three independent experiments; (**B**) Western blot using Vero cells infected with either rMP-12, Δ2 + 3, or the AUG2-M mutants (an MOI of 1). The 78 kD and Gn were detected by mouse anti-Gn monoclonal antibody (4D4). The N proteins were detected using mouse anti-RVFV polyclonal antibody. β-actin is shown as sample loading controls; (**C**) plaque phenotypes of rMP-12, Δ2 + 3, or the AUG2-M mutants in VeroE6 cells. Small (S) and large (L) plaques are shown with arrows. The diameters (mm) of small and large plaques were measured (*n* = 10 per sample), and the average and standard errors are shown in the graph.

Statistical analysis was performed using GraphPad Prism version 6.05. For the gLuc/cLuc values normalized to parental construct value in Figure 2 and Figure 3, or virus titers in Figure 4, arithmetic means of log_10_ values were analyzed by one-way ANOVA followed by Tukey’s multiple comparisons test.

## 3. Results

### 3.1. The Gn/gLuc Precursor-1 Does Not Efficiently Generate Gn/gLuc Fusion Proteins

293 cells were co-transfected with pSV40-CLuc (transfection control), and pCAGGS-PreGn-gLuc-SF or the AUG mutant plasmids. At 36 h post transfection, cell lysates were collected and the intracellular expression of Gn/gLuc proteins or precursor proteins was analyzed by Western blot using anti-Flag antibody. Culture supernatants were used to measure the level of secreted extracellular Gn/gLuc fusion proteins by reporter assay. The cLuc protein encodes an intrinsic signal peptide and is secreted into the secretory pathway after expression. Thus, secreted cLuc served as a control to measure the secretion of the Gn/gLuc fusion proteins from transfected cells. Western blot using anti-Flag antibody was performed to confirm the expression of precursor proteins and Gn/gLuc fusion proteins (Figure 2A). As expected, the Gn/gLuc precursor-1 disappeared after the abolishment of the 1st AUG. Similarly, the Gn/gLuc precursor-2 disappeared when the 2nd AUG was abolished. When the 2nd AUG was abolished, the Gn/gLuc precursor-3 appeared (*i.e.*, Δ2 or Δ1 + 2). The Gn/gLuc precursor-4 and -5 were indistinguishable from the cleaved Gn/gLuc fusion protein based on band migrations. When the 1st, 2nd, 3rd, 4th, and 5th AUGs were abolished, no Gn/gLuc fusion proteins were detected. On the other hand, when the 2nd, 3rd, 4th, and 5th AUGs were abolished, the band intensity of the Gn/gLuc fusion protein was largely decreased but still detectable in the Western blot. Next, both gLuc and cLuc activities in culture supernatants were measured. We calculated the ratio of gLuc to cLuc, and the value was normalized to that of parental pCAGGS-PreGn-gLuc-SF (Figure 2B). When the 1st, 2nd, 3rd, 4th, and 5th AUGs were all abolished, no gLuc activity (0.79% compared to parental 100%) could be measured in the supernatant. On the other hand, when the 2nd, 3rd, 4th, and 5th AUGs were abolished, a decreased level of gLuc activity (7.7% compared to parental 100%) was detected in the supernatant. These results indicated that the precursor protein synthesized from the 1st AUG can also generate Gn, though at a decreased level.

### 3.2. Precursor-2 Plays a Major Role in Gn/gLuc Expression

We further analyzed the relative secretion of the cleaved gLuc using various AUG mutants (Figure 2B). The 3rd and 4th AUGs (Δ3 + 4), the 3rd, 4th, and 5th AUGs (Δ3 + 4 + 5); or the 4th and 5th AUGs (Δ4 + 5) were abolished, the gLuc activity was 142%, 104%, or 146%, respectively, compared to the parental plasmid (100%). It indicates that abolishment of the 3rd and the 4th AUGs, that of the 4th and the 5th AUGs, and that of the 3rd, the 4th, and the 5th AUGs, do not result in a decrease of relative gLuc activity, in the presence of the 1st and the 2nd AUGs. The individual abolishment of the 1st (Δ1), 2nd (Δ2), 3rd (Δ3), or 4th AUG (Δ4), resulted in relative gLuc activities of 121%, 32%, 85%, or 72%, respectively, compared to the parental plasmid (100%) (Figure 2B). The Δ2 mutant generated significantly lower gLuc activity than the Δ4 + 5 mutant, the Δ1 mutant, or the Δ3 mutant. Thus, the abolishment of the 2nd AUG significantly affected the gLuc activity. When the 1st and 2nd AUGs (Δ1 + 2); the 1st, 2nd, and 3rd AUGs (Δ1 + 2 + 3); or the 1st, 2nd, 3rd, and 4th AUGs (Δ1 + 2 + 3 + 4) were concomitantly abolished, the gLuc activity was 74%, 13%, or 15%, respectively, compared to the parental plasmid (100%). The gLuc activities of the Δ1 + 2 + 3 mutant and the Δ1 + 2 + 3 + 4 mutant were significantly lower than that of the Δ1 + 2 mutant. Furthermore, when the 2nd and 3rd AUGs (Δ2 + 3); the 2nd, 3rd, and 4th AUGs (Δ2 + 3 + 4); or the 2nd, 3rd, 4th, and 5th AUGs (Δ2 + 3 + 4 + 5) were abolished, the gLuc activity was 31%, 18%, or 8%, respectively, compared to the parental plasmid, and all of which were significantly lower than that of the Δ4 + 5 mutant. Although it was not statistically significant, the differences of gLuc activity between the Δ1 + 2 + 3 (13%) and Δ2 + 3 mutants (31%), or between the Δ1 + 2 + 3 + 4 (15%) and Δ2 + 3 + 4 (18%), indicate that the precursor from the 1st AUG slightly increases gLuc activity. Taken together, these results indicate that the secretion of Gn/gLuc fusion proteins into the culture supernatant occurs efficiently through the Gn/gLuc precursor-2, while the Gn/gLuc precursor-3 can serve as a surrogate of Gn/gLuc precursor production in the absence of AUG-2. However, it was unclear why the secretion of the Gn/gLuc fusion protein from the precursor-4 or 5 was lower than those of precursor-2 or 3.

### 3.3. The Viral Untranslated Region Sequence, Upstream of the 4th or 5th AUG, Affects Efficient Generation of Gn/gLuc Fusion Proteins

Spik *et al.* previously showed that the cloned open reading frame (ORF) of Gn-Gc (starting from the 4th AUG), without an upstream viral untranslated region (UTR), expresses slightly higher Gn/Gc than the cloned ORF of the NSm-Gn-Gc precursor (starting from the 2nd AUG) [38]. Thus, we suspected that the decrease in the gLuc activity from the Gn/gLuc precursor-4 or -5 occurred due to the presence of the UTR upstream of the 4th or 5th AUG. Therefore, we truncated the upstream viral UTR sequence from pCAGGS-PreGn-gLuc-SF (Figure 3A). The plasmids, AUG2-M, AUG3-M, AUG4-M, or AUG-5-M, generate the Gn/gLuc precursor-2, -3, -4, or -5, respectively, and share the common UTR sequence (5′-ACACAAAGACGGUGCACGAGAUG (initiation codon is underlined)). Each plasmid also lacks downstream initiation codons, to prevent the generation of additional precursor proteins. This abolishment allowed us to analyze the role of a single precursor protein in the production of the Gn/gLuc fusion protein. Using those plasmids, we analyzed the secretion of the Gn/gLuc fusion proteins into the culture supernatant. Surprisingly, AUG2-M had 282% gLuc activity, which was significantly higher than that of AUG3-M, AUG4-M, or AUG5-M. On the other hand, the AUG3-M had significantly lower gLuc activity (66%) than that of the AUG4-M (116%) or the AUG5-M (92%). The difference of gLuc activity between AUG4-M and AUG5-M was marginally significant. The results indicated that precursor-3, produced from the AUG3-M, is less efficient than precursor-4 from the AUM4-M in the secretion of the Gn/gLuc fusion protein. Since the gLuc activities of Δ1 + 2 + 3, and Δ1 + 2 + 3 + 4 mutants were 13%, and 15% (Figure 2B), respectively, the viral UTR upstream of the 4th or 5th AUG affects the translation efficiency of precursor-4 or 5. Furthermore, the AUG2-M mutant increased the secretion of the Gn/gLuc fusion protein, compared to the Δ1 mutant, indicating that viral UTR upstream of the 2nd AUG also affects the translation efficiency of precursor-2.

### 3.4. The rMP-12 Encoding the AUG2-M Mutation or the rMP-12 Encoding the Δ2 + 3 Mutation Replicate Less Efficiently Than Parental rMP-12

We, next, characterized the viral phenotypes caused by modification of the M-segment precursors. The reporter assay results indicated that the AUG2-M mutant plasmid generates Gn proteins efficiently, due to a lack of upstream viral UTR sequence. Thus, we aimed to test whether the recombinant MP-12 encoding the AUG2-M mutations in the preglycoprotein coding region can replicate more efficiently than other mutants. However, the modification of preglycoprotein region also affects the expression of the 78 kD, NSm, or NSm’. The AUG2-M mutant does not encode the 78 kD and NSm’ proteins, but encodes the NSm protein with 3 mutations (Met to Leu, at the 3rd, 4th, and 5th AUGs). For comparison, we also analyzed the Δ1 mutant (lacking the 78 kD, but still encoding NSm and NSm’), Δ1 + 2 mutant (lacking 78 kD and NSm, but encoding NSm’), and the Δ2 + 3 mutant (encoding 78 kD, but lacking NSm and NSm’). Those constructs were predicted to express Gn protein less efficiently than the AUG2-M mutant construct, based on reporter assay result. As a control, the Δ4 + 5 mutant was analyzed, which produces the default precursors from the 1st and 2nd AUGs.

We, first, analyzed the replication of rMP-12 mutants (the AUG2-M, Δ1, Δ1 + 2, Δ2 + 3, or Δ4 + 5) at a multiplicity of infection (MOI) of 0.15. All the rMP-12 mutants replicated efficiently in Vero cells, while the rMP-12 encoding the Δ2 + 3 mutation, or that encoding the AUG2-M mutation replicated slightly more slowly than the others (Figure 4A). The arithmetic means of log_10_ titers of AUG2-M mutant at 48 and 72 hpi were 7 and 2 times lower than those of the Δ4 + 5 mutant at 48 and 72 hpi, respectively (*p* < 0.05). On the other hand, the arithmetic means of log_10_ titers of Δ2 + 3 mutant were 6 and 2 times lower than those of the Δ4 + 5 mutant at 48 and 72 hpi, respectively, and the differences were not statistically significant. These two mutants, AUG2-M and Δ2 + 3, were further analyzed at a higher MOI infection (1 MOI). After virus infection at 37 °C for 1 h, Vero cells were washed 6 times with media, and the cell lysates were collected at 3, 4, 5, 6, 7, and 8 hpi. Western blot analysis showed that parental rMP-12 generated a detectable level of 78 kD at 6, 7, and 8 hpi, and NSm-Gn at 7 and 8 hpi (Figure 4B). On the other hand, 78 kD was not synthesized from the AUG2-M mutant, and the NSm-Gn was not made from the Δ2 + 3 mutant. Viral RNA replication most likely started between 5 and 6 hpi, as there was an increase of all viral proteins at 6 hpi. The parental rMP-12 made plaques with heterogeneous sizes (2.6 to 5.7 mm in diameter). The Δ2 + 3 mutant made smaller plaques than parental rMP-12 ranging from 0.9 to 2.3 mm in diameter (Figure 4C). On the other hand, the AUG2-M made the intermediate sized plaques between the Δ2 + 3 and parental rMP-12 (1.7 to 4.5 mm in diameter). Taken together, these results indicate that rMP-12 encoding the AUG2-M mutation replicates less efficiently than Δ4 + 5 mutant, though the NSm-Gn-Gc precursor made from the AUG2-M mutant was predicted to generate more Gn than Δ4 + 5 mutant. Thus, the discrepancy of reporter assay and virus replication efficiency suggested a role of 78 kD, NSm, or NSm’ in the downstream expression of Gn.

## 4. Discussion

A live-attenuated RVFV vaccine, MP-12, is conditionally licensed for veterinary use in the U.S. Though the MP-12 vaccine is safe and efficacious [19,20,21,22,39], the vaccine lacks a marker for DIVA (differentiation of infected from vaccinated animals). Reverse genetics is a useful tool to generate RVFV lacking either the 78 kD or NSm proteins. The Δ78 kD or ΔNSm mutant can be made by the abolishment of the 1st or the 2nd AUG, respectively. An introduction of a DIVA marker in the M-segment can be made by truncating the 78 kD/NSm coding region ranging from the 1st AUG to the downstream of the 3rd AUG, while leaving a short UTR upstream of the 4th AUG. The rMP12-ΔNSm21/384 (similar to our AUG4-M mutant plasmid), which lacks both 78 kD and NSm expression, showed a similar immunogenicity and efficacy with parental MP-12 [40,41]. However, without knowing the role of each AUG or UTR in the Gn/Gc expression or virion productions, the impact of alterations of the preglycoprotein coding region on the virological phenotype cannot be predicted. In this study, we analyzed the effect of AUG abolishment or an in-frame deletion of viral UTR sequence upstream of the AUG on the Gn expression levels using a quantitative reporter assay system. Subsequently, we also characterized the virological phenotypes of representative AUG mutants. Initially, we hypothesized that increased expression levels of Gn/Gc would increase progeny virus titer. However, that assumption was not correct, and the results showed an unexpectedly complex regulation of viral progeny production through Gn and Gc. As increased production of Gn did not lead to an increase in virus production, regulation of viral production may be regulated at a later step in the viral life cycle.

The reporter assay expressing the Gn/gLuc fusion protein downstream of the RVFV preglycoprotein coding region is useful to measure the level of cleaved Gn/gLuc from the precursor proteins translated from the 1st, 2nd, 3rd, 4th, or 5th AUG. Since the Gn/gLuc fusion protein does not encode the Golgi retention signal, the protein is secreted out from transfected cells without accumulation in the Golgi. Thus, the relative reporter activities in the culture supernatants indicate the efficiency of precursor expression, and subsequent cleavage of the precursor protein. We confirmed that the plasmid lacking the 2nd, 3rd, 4th, and 5th AUGs (Δ2 + 3 + 4 + 5) still generates a small amount of the Gn/gLuc fusion protein and a detectable gLuc activity in the culture supernatant. The 78 kD protein encodes a signal sequence at the N-terminus [28], and the 78 kD-Gc precursor is synthesized in the ER membrane. The second signal sequence for 78 kD protein, which is located between the NSm and Gn coding region, may not be efficiently recognized by signal peptidase, which may be hindered by the folding of ectodomain in the ER lumen. Indeed, we could not rescue the rMP-12 encoding the Δ2 + 3 + 4 + 5 mutation in the M-segment, most likely due to such low expression of Gn from the 78 kD-Gc precursor. The Δ2 showed decreased secretion of the Gn/gLuc fusion proteins (32%) in culture supernatants. However, the Δ1, Δ3, or Δ4 mutant still efficiently secreted the Gn/gLuc fusion proteins (121%, 85%, or 72%, respectively). In addition, in the presence of the 1st and 2nd AUG, the abolishment of the 3rd, 4th, or 5th AUG (Δ3 + 4 + 5) did not affect the gLuc activity (104%). Thus, the precursor-2, produced by AUG 2, plays a role in the production of the Gn/gLuc fusion protein. Since the Δ1 + 2 mutant still expresses relatively high gLuc activity (74%), compared to the Δ1 + 2 + 3 mutant (13%), the precursor-3, but not precursor-4, or 5, serves as an efficient surrogate of precursor-2 in the production of the Gn/gLuc fusion protein. Thus, our results indicated that the NSm-Gn-Gc precursor plays a default role, and the NSm’-Gn-Gc precursor plays a surrogate role, in the expression of the Gn protein.

Although we introduced mutations to abolish specific AUGs, this approach did not address the effect of long viral UTR upstream of AUG in the translation efficiency of Gn. Relative gLuc activities of the Δ1 + 2 + 3 or Δ1 + 2 + 3 + 4 mutants were low. To address this concern, we generated additional reporter constructs encoding the 2nd, 3rd, 4th, or 5th AUG, without upstream viral UTR sequences (AUG2-M, AUG3-M, AUG4-M, or AUG5-M, respectively). We also abolished downstream AUGs, to prevent the expression of more than one precursor protein. The AUG4-M (116%), and AUG5-M (92%) constructs showed increased gLuc activity, compared to Δ1 + 2 + 3 (13%), and Δ1 + 2 + 3 + 4 (15%) mutant plasmids. Thus, the deletion of the UTR upstream of the 4th or 5th AUG improved the secretion of the Gn/gLuc fusion protein from precursor-4, or 5, respectively. Those results indicated that an in-frame deletion of UTR sequence increases the expression of Gn/Gc from the precursor made from the 4th or 5th AUG.

A limitation of this reporter assay is the lack of natural viral assembly and budding from the Golgi. The results from reporter assay predict the expression levels of Gn proteins from mRNA. However, RVFV Gn encodes a Golgi retention signal at the C-terminus, and co-localizes with Gc to form heterodimers [30,42,43]. Gn and Gc are assembled with the viral ribonucleocapsid, bud from the Golgi, and out of the cell. On the other hand, little is known about the functions of 78 kD, NSm, or NSm’ in the assembly process. We generated recombinant rMP-12 encoding mutations in the preglycoprotein coding region (Δ1, Δ1 + 2, Δ2 + 3, Δ4 + 5, or AUG2-M). Our study showed that the rMP-12 encoding the AUG2-M mutations replicated slightly less efficiently than parental rMP-12 in Vero cells, despite having increased Gn expression. The AUG2-M does not make 78 kD and NSm’, but encodes NSm having Met-to-Leu substitutions at the 3rd, 4th, and 5th AUGs. The Met-to-Ala substitution at the 3rd AUG is known to decrease the migration of NSm protein [18], indicating the occurrence of posttranslational modification of NSm. We assume that NSm and NSm’ play a role at a later step of viral protein synthesis: For example, viral assembly process. The Δ2 + 3 mutant encode neither NSm nor NSm’ but still generates the 78 kD protein [18]. In our study, this mutant also showed relatively inefficient replication kinetics in Vero cells, although it was not statistically significant. The NSm of Bunyamwera virus (BUNV: genus *Orthobunyavirus*) serves as a scaffold to form a “viral tube” structure to facilitate the assembly of the ribonucleocapsid with the Gn/Gc complex at the peripheral Golgi, and a lack of NSm reduce virus production by 10 to 100-fold [44]. The 78 kD protein may also play a role in viral replication. Kreher *et al.* showed that the AUG (Met) to GCG (Ala) mutation at the 1st AUG (Δ1), at the 1st and 2nd AUGs (Δ1 + 2), or at the 1st, 2nd, and the 3rd AUGs (Δ1 + 2 + 3) led to the emergence of RVFV mutant encoding a new AUG upstream of the original 1st AUG, during 5 serial passages in mammalian cells [18]. This new AUG can generate a 78 kD-Gc-like precursor protein, in addition to the NSm-Gn-Gc precursor. Previous studies indicated that the 78 kD plays a major role in viral dissemination in mosquito vectors, while NSm and NSm’ contribute to the RVFV propagation in vertebrate cells [18]. However, little is known about the role of 78 kD, NSm, or NSm’ proteins in viral replication, and further studies will be required to elucidate the mechanisms.

## 5. Conclusions

This study showed that, not only the abolishment of AUG, but also the truncation of viral UTR, affects the expression of Gn protein by the RVFV M-segment. Increased production of Gn did not lead to an increase in virus production, and thus, regulation of viral production may be further regulated at a later step in the viral life cycle.

## Abbreviations

The following abbreviations are used in this manuscript:

### Abbreviations

RVF: Rift Valley fever
RVFV: Rift Valley fever virus
L-segment: Large-segment
M-segment: Medium-segment
S-segment: Small-segment
DIVA: Differentiation of infected from Vaccinated animals
gLuc: *Gaussia* luciferase
cLuc: *Cypridia* luciferase
PKR: dsRNA-dependent protein kinase
IFN: Interferon
FBS: Fetal bovine serum
BHK: Baby hamster kidney
MOI: Multiplicity of infection
SDS-PAGE: Sodium dodecyl sulfate-polyacrylamide gel electrophoresis
CAG promoter: The cytomegalovirus early enhancer/chicken β actin promoter
UTR: Untranslated region
